# Polarization-sensitive optical projection tomography for muscle fiber imaging

**DOI:** 10.1038/srep19241

**Published:** 2016-01-11

**Authors:** Mengjie Fang, Di Dong, Chaoting Zeng, Xiao Liang, Xin Yang, Alicia Arranz, Jorge Ripoll, Hui Hui, Jie Tian

**Affiliations:** 1 Key Laboratory of Molecular Imaging, Institute of Automation, Chinese Academy of Sciences, Beijing 100190, China; 2 Beijing Key Laboratory of Molecular Imaging, Beijing 100190, China; 3 Department of Hepatobiliary Surgery, Zhujiang Hospital, Southern Medical University, Guangdong 510282, China; 4 Center for Molecular Biology “Severo Ochoa” (CSIC-UAM), Madrid 28049, Spain; 5 Department of Bioengineering and Aerospace Engineering, Universidad Carlos III of Madrid, Madrid 28911, Spain

## Abstract

Optical projection tomography (OPT) is a tool used for three-dimensional imaging of millimeter-scale biological samples, with the advantage of exhibiting isotropic resolution typically in the micron range. OPT can be divided into two types: transmission OPT (tOPT) and emission OPT (eOPT). Compared with eOPT, tOPT discriminates different tissues based on their absorption coefficient, either intrinsic or after specific staining. However, it fails to distinguish muscle fibers whose absorption coefficients are similar to surrounding tissues. To circumvent this problem, in this article we demonstrate a polarization sensitive OPT system which improves the detection and 3D imaging of muscle fibers by using polarized light. We also developed image acquisition and processing protocols that, together with the system, enable the clear visualization of muscles. Experimental results show that the muscle fibers of diaphragm and stomach, difficult to be distinguished in regular tOPT, were clearly displayed in our system, proving its potential use. Moreover, polarization sensitive OPT was fused with tOPT to investigate the stomach tissue comprehensively. Future applications of polarization sensitive OPT could be imaging other fiber-like structures such as myocardium or other tissues presenting high optical anisotropy.

Optical projection tomography (OPT) has become an important imaging tool in biomedical research^1^,^2^,^3^,^4^. Using OPT, biological samples of up to 1 cm can be observed in-toto with a resolution of microns. Up-to-date, OPT modalities comprised transmission OPT (tOPT) and emission OPT (eOPT). In tOPT, collimated light penetrates the sample and is collected by the camera as a projection view of the sample, and then multi-view projection views spanning the sample are used to reconstruct the 3-Dimensional (3D) distribution of light absorption coefficients in the sample. On the other hand, in eOPT, the fluorescent light emitted from the sample is collected by means of appropriate filters in order to obtain a 3D image of fluorophore distribution. Optical clearing protocols are extensively used in *ex-vivo* imaging to eliminate the internal scattering^5^,^6^,^7^,^8^.

3D imaging of muscle fibers is an interesting tool for studies in oncology, developmental biology, and histopathology^9^,^10^,^11^,^12^. For example, leiomyosarcoma, a cancer affecting smooth muscle can be analyzed by investigating changes in the texture and shape of the muscle fibers^9^. In addition, muscle atrophy has been suggested as a prognostic factor in cancer^10^. In this regard, mesoscopic imaging methods such as OPT could provide new procedures for studying muscle fibers in different diseases and models. In practice, due to the lack of contrast between individual fibers it is currently not possible to distinguish muscle fibers from its surrounding tissues by tOPT, requiring fluorescent specific labeling and the use of high resolution eOPT. We believe that including polarized measurements in tOPT would enhance the contrast between muscle fibers allowing 3D imaging in a simple and label-free manner.

In this article, the use of polarized light was implemented into a tOPT to analyze its potential use in the detection of muscle fibers. Polarized light has previously been used for imaging of biological samples^13^,^14^,^15^,^16^,^17^,^18^. For example, polarization-sensitive optical coherence tomography has allowed the observation of superficial tissues with small fields of view^15^. Using light polarization, information may be obtained from deeper tissues^16^, detecting special optical properties such as birefringence^17^. In addition, polarized light imaging with mechanical sectioning allowed investigating deep fiber structures, with the drawback that the tomographic imaging is invasive and destructive^18^. In previous work^19^, light polarization was also employed in OPT for mesoscopic imaging of scattering, concentrating on the tomographic reconstruction of scattering coefficients. However, up to date polarization-sensitive OPT has not been used to observe biological specimens presenting optical anisotropy, such as muscle fibers. Therefore, a mesoscopic imaging method for 3D imaging of anisotropic tissues is still in great demand.

To circumvent this problem, in this article we propose a polarization-sensitive OPT system together with an image acquisition protocol and an image reconstruction and processing scheme for muscle fiber imaging. In contrast with current OPT methods, polarization-sensitive OPT may differentiate anisotropic tissues such as muscle fibers based on their birefringence properties. The result shown here demonstrates that polarized light enhanced the detection of muscle fibers not only with our custom OPT system, but also when applied to a modified commercial microscope, underlying the validity and ease of translation of polarization-sensitive OPT. In the following paragraphs, we describe the use of polarized light on transparent muscle fiber imaging and our system, presenting the image acquisition protocol as well as the image reconstruction and processing schemes before presenting our concluding remarks.

## Methods

### Polarization-sensitive OPT System

**Figure 1:**
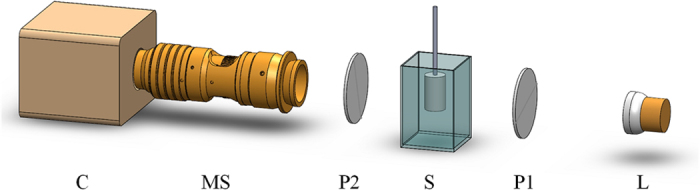
Schematic of the experiment setup. P1: the first polarizer (the polarizer). S: sample. P2: the second polarizer (the analyzer). MS: microscope system. C: EMCCD camera. The strips embedded in the two polarizers represent their polarized directions.

In order to observe the muscle fibers in 3D, we built a polarized OPT system by adding a pair of polarizers to our previous OPT system^20^,^21^,^22^. The experimental setup of our system is shown in Fig. 1. A linear polarizer (GCL-050004, Daheng Optics, Beijing, China) placed in front of a partially collimated white light source was used to provide homogenous linearly polarized light. After traversing the sample, the degree of polarization was measured by an EMCCD camera (iXon DV885, Andor Technology, Belfast, U.K.) coupled to a telecentric lens (Leica, Bensheim, Germany) by the use of a second linear polarizer (GCL-050004, Daheng Optics, Beijing, China). Through the relative rotation of both polarizers, the angle of polarization could be determined. In the system, we always kept the polarization direction of the two polarizers perpendicular to each other and rotated them simultaneously so as to remove the direct incident light.

### Sample Preparation

Sections of the diaphragm from a healthy nude mouse were obtained and imaged fresh in a commercial stereo microscope (M205 FA, Leica, Bensheim, Germany). While preparing the sample we made sure the central tendon and the muscle fiber surrounding the tendon were present. After taking a picture of the diaphragm on the commercial stereo microscope, it was optically cleared by using a BABB solution (1:2 ratio of Benzyl alcohol and Benzyl benzoate), after fixing with 4% paraformaldehyde at 4 °C overnight and dehydrated by immersing successively in, 25, 50, 75 and 100% methanol at room temperature 2 hours per step. Once dehydrated the samples was kept in BABB at room temperature for 48 h.

Two samples of gastric wall from Sprague Dawley (SD) rats were fixed and trimmed after dissection. One was dehydrated and optically cleared using the BABB solution described above. The other sample was fluorescently labeled with the large tissue immunolabeling method iDISCO^6^. The mouse monoclonal smooth muscle actin (SMA) antibody (Boster, China) and Alexa Fluor 488 -conjugated secondary antibodies (Life Technologies) were applied to label the muscles. The whole labeling step took two weeks due to the large size of the sample. After immunolabeling the sample was optically cleared by using the BABB solution.

All animals were purchased from the Department of Experimental Animals, Peking University Health Science Center. All experimental protocols were approved by the Institutional Animal Care and Use Committee (IACUC) at Peking University, and all the methods were carried out in accordance with the approved guidelines.

### Image Reconstruction Scheme

When imaging with polarization-sensitive OPT, the direction of the incident polarized light will be partly changed due to the muscle fiber’s birefringent properties. Note that, besides the muscle fibers, other anisotropic tissues will also present birefringence, caused by the refractive index difference between fibers and their surrounding extracellular matrix (form birefringence) or between different chemical groups of proteins (intrinsic birefringence), along a particular axis (i.e. this change of index of refraction is anisotropic)^23^. The birefringence properties in optically cleared samples are mainly caused by the regular fiber structures, since optical clearing method homogenizes the refractive indices, reducing opacity and the intrinsic birefringence. In addition, the muscle fibers are tight together in bundles, exhibiting high birefringence. Therefore in polarization-sensitive OPT, the direction of incident polarized light will be partly changed after passing through the muscle fibers, being detected with maximum intensity with the direction of the analyzer coincides with the direction of the fiber structure.

Since the polarized light is mainly changed by fiber bundles in the cleared samples, the intensity of the polarized image detected by the camera can be described by the formula which describes the transmission of polarized light in the medium of birefringent fibers^14^,^18^,^24^:


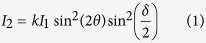


where 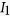 is the light intensity after passing through the sample, and 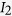 is the light intensity after passing through the analyzer (P2 in Fig. 1). 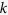 is an attenuation coefficient which depends on the light absorption in the light transmission path. 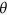 is the minimal angle between the projection of fiber orientation in the polarizer plane and polarized directions of the two linear polarizers. 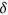 is a resultant phase shift, and it can be formulated as:


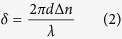


where 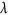 is the wavelength of the polarized light. 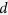 is the thickness of birefringent tissue, and 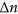 is the difference between the refractive indices encountered by the two constituent beams of polarized light, which are parallel to and perpendicular to the fiber bundle respectively. Note that the samples in this paper were very thin and nearly transparent after optical clearing by BABB. Moreover, the sample was embedded in an agarose cylinder being the attenuation coefficient practically homogeneous for different measurement angles. Therefore, the attenuation was not taken into account and the attenuation coefficient *k* was set at a fixed value. However, when imaging dense and highly irregular tissue such as bones and blood clots, the attenuation coefficient difference should be considered for improved quantitative imaging. As shown in the formulas above, there is no positive correlation between the polarized light intensity through the analyzer and the refractive indices or the thicknesses of the birefringent tissues. Furthermore, the light intensity detected by the camera changes following the system polarized angle with a periodic interval of 90°. Thus, there will be four intensity peaks spanning 360°.

As 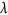 has a direct effect on the light intensity of transmitted light passing through the analyzer, a white light source with a wide wavelength range was used, in order to provide roughly the wavelength independent light intensities at different system polarized angles. Moreover, in order to achieve a balance between experimental complexity and image quality, 3 polarized images with an interval of 30° between them were chosen to generate a fused projection view in our experiments, selecting the maximum intensity value of the three images for each pixel.

In order to obtain a 3D image, additional to the 3 polarization measurements multi-view projections need to be acquired. Typically, 360 projection views around the sample with an interval angle of 1° are enough for an accurate 3D OPT reconstruction. In our particular case 360 polarized images were collected by rotating the sample and recording the images simultaneously at a fixed system polarized angle, repeating this procedure for a total of 3 different polarization angles. After the data collection, three polarized image stacks were merged into one projection view stack by maximum intensity projection. A filtered back-projection (FBP) method with parallel acceleration developed by our group for eOPT^21^ was used for 3D reconstruction.

## Results and Discussion

### Polarization Sensitive Imaging

**Figure 2:**
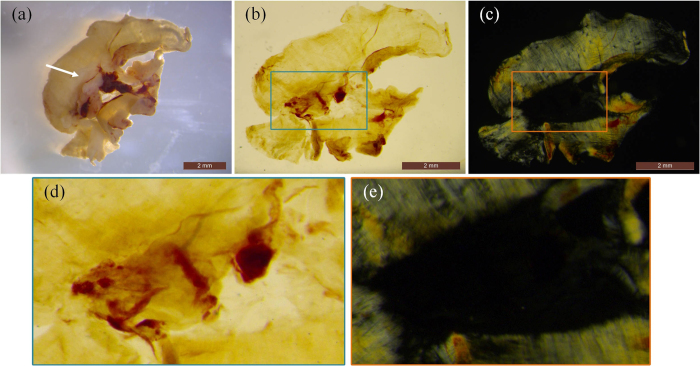
The muscle fibers after clearing is observed by polarized light. (**a**) a photo of the diaphragm sample before the clearing process. The projection view of the cleared diaphragm captured through (**b**) non-polarized light and (**c**) polarized light. Scale bar: 2 mm. (**d,e**) zoomed images in the boxes of (**b,c**).

In order to test possibility of measuring the birefringence properties of tissue with polarized light, we first show a result of mouse diaphragm from a commercial microscope to check whether the muscle fibers after clearing could be observed by polarized light. As shown in Fig. 2(a), a section of the diaphragm from a healthy nude mouse was obtained and imaged fresh in a commercial stereo microscope (M205 FA, Leica, Bensheim, Germany). The diaphragm consisted of two parts: the central tendon in the middle (arrowhead in Fig. 2(a)) and the muscle fiber surrounding the tendon. After taking a picture of the diaphragm, it was optically cleared (see Materials and Methods – Sample preparation). The projection view of the transparent diaphragm tissue with non-polarized illumination is shown in Fig. 2(b,d) in which the muscle fibers could not be distinguished from the aponeurosis due to their similar light absorption coefficients. In contrast, after illuminating with the polarized light and detecting with a perpendicular direction analyzer, the aponeurosis disappeared but the muscle fibers remained in the polarized image (shown in Fig. 2(c,e)). Moreover, the texture of the muscle fiber was obvious in the polarized image.

The contrast presented by the muscle fibers in the polarized image is based on the phenomenon that polarized direction of the light is changed when penetrating the birefringent medium like the muscle fibers. Therefore, by filtering the sample images with an analyzer whose polarized direction is perpendicular to the original light, the muscle fibers can be specifically observed through the deflection light. It should be noted that the prominent detection of muscle fibers also benefits from the clearing process which dramatically decreases light scattering. Otherwise the presence of scattering would not only blur the image but also change light polarization^19^,^25^.

**Figure 3:**
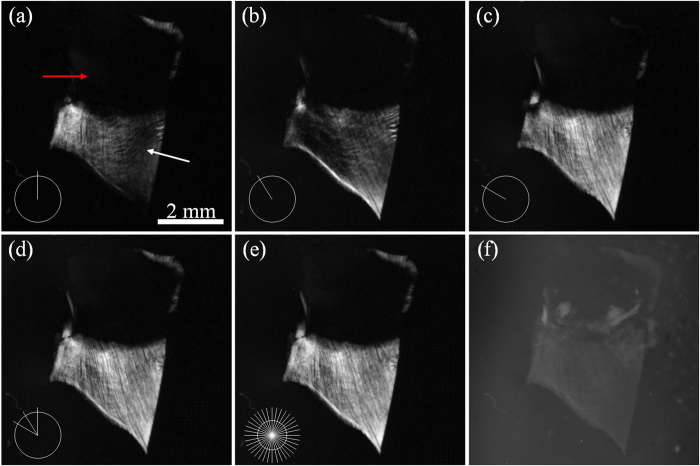
3 directions of polarization enable an enough detection of the muscle fibers. (**a–c**) the polarized images of a cleared diaphragm captured at 3 different system polarized angles with an interval angle of 30°. The red arrow shows the central tendon and the white arrow presents the muscle fibers. (**d**) the fused image of (**a–c**). (**e**) the fused image of 36 polarized images spanning 360° with an interval angle of 10°. The inset in the bottom left corner of each image represents the relative system polarized angles. (**f**) the projection view of the sample in traditional tOPT. This image is inverted for improved visualization.

Beside the results from the commercial system, our polarization-sensitive OPT was also used to observe a transparent thin diaphragm. As shown in Fig. 3(a–c), three polarized images of the diaphragm with an interval system polarized angle of 30° demonstrated differences in image intensities and textures. Note that, polarization-sensitive OPT at a fixed system polarized angle is sensitive to muscle fibers in certain direction, and therefore a single polarized image is not enough for comprehensive investigation of the fibers. Therefore, in order to provide a robust image, independent on the polarization angle detected a fused image of several polarization angles should be obtained, noting that a 90 degree coverage is representative of a full 360° rotation. In our experiments, 3 polarized images with an interval of 30° between them were used to generate a fused projection view. Fig. 3(d,e) present the fused projection views of 3 and 36 polarized images respectively, showing no significant differences between them. The projection view fused by 3 different polarized images covered 99.1% of the muscle fiber region of the fusion view by 36 images, while the three polarized images covered only 66.9%, 76.0% and 92.7% of the region respectively. Furthermore, as shown in Fig. 3(d), the muscle fibers were enhanced in the fused projection view, and the texture was clearer. For purposes of comparison, a projection view obtained by tOPT is shown in Fig. 3(f), in which the textures of the muscle fiber were indistinguishable and image intensity of the sample was weak due to the thin thickness of the diaphragm. It should be noted that this image has been inverted for better comparison with the polarized images which have a dark background. The following tOPT images were all inverted for improved visualization. The signal-to-background ratios (SBR), a ratio of the mean pixel intensity in the region of interest to the one in the region of background, were approximately 1.52 in Fig. 3(f) and 11.64 in Fig. 3(d), showing the polarization-sensitive OPT greatly enhanced SBR of the muscle fibers. The reason is that the light without depolarization was blocked by the analyzer and background signals were severely suppressed.

### 3D Imaging of Tissue Anisotropy

**Figure 4:**
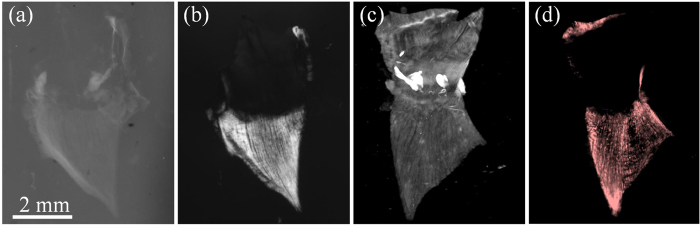
The polarization-sensitive OPT provides a specific imaging results of the muscle fibers. (**a**) a projection view of the diaphragm in tOPT. This image is inverted for improved visualization. (**b**) the corresponding fused projection view in polarized OPT. (**c,d**) the volume rendering of 3D results of tOPT and polarization-sensitive OPT with maximum intensity projection.

The polarization-sensitive OPT was further applied to mouse diaphragm tissue for 3D imaging. The 3D results are shown in Fig. 4(d) where the muscle fibers as well as the texture are clearly visible. In contrast, the central tendon and muscle fiber were not easy to differentiate in tOPT (see Fig. 4(c)) due to the homogenization of refractive indices and absorption coefficients.

**Figure 5:**
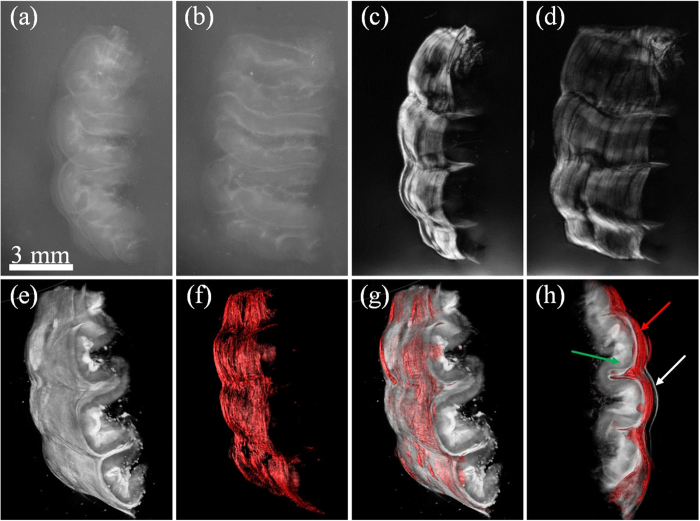
When combined with tOPT, the polarization-sensitive OPT provides more details of the anatomic information. (**a,b**) two projection views of the gastric wall in tOPT. These two images are inverted for improved visualization. (**c,d**) two fused projection views in polarization-sensitive OPT. (**e,f**) the volume rendering results of tOPT and polarization-sensitive OPT. (**g,h**) the volume rendering of the fusion image of polarization-sensitive OPT and tOPT. Green arrow: the mucosa and the submucosa. Red arrow: the muscularis externa. White arrow: the serosa.

As seen in Fig. 4, while polarization-sensitive OPT generates high contrast images of muscle fibers, tOPT offers very useful anatomical information, clearly pointing out the advantage of combining both approaches. In order to showcase this fact, we imaged a thick sample of gastric wall from a healthy rat, with polarization-sensitive OPT and tOPT. In Fig. 5 a volume render of each approach while measured independently and a combination of both is presented, clearly highlighting the muscle fibers while providing the underlying anatomical structure. Typically, the gastric wall from outside to inside is divided into four layers, namely, the mucosa, submucosa, muscularis externa, and serosa. As shown in Fig. 5(a,b) muscle fiber was not easy to differentiate in tOPT, while the polarization-sensitive OPT highlighted the fiber structures in the muscularis externa (shown in Fig. 5(c,d)). Moreover, the 3D volume rendering results of tOPT and polarization-sensitive OPT in Fig. 5(e,f) illustrated different features of the gastric wall. Finally, the 3D volumes of polarization-sensitive OPT and tOPT were merged together to show an accurate position of the muscularis externa. The volume visualization of the fusion images were shown in Fig. 5(g,h) in which the muscularis externa was clearly distinguished from the serosa and submucosa. These results showcase the fact that polarization-sensitive OPT fused with tOPT has great potential in studies involving anisotropic tissues.

**Figure 6:**
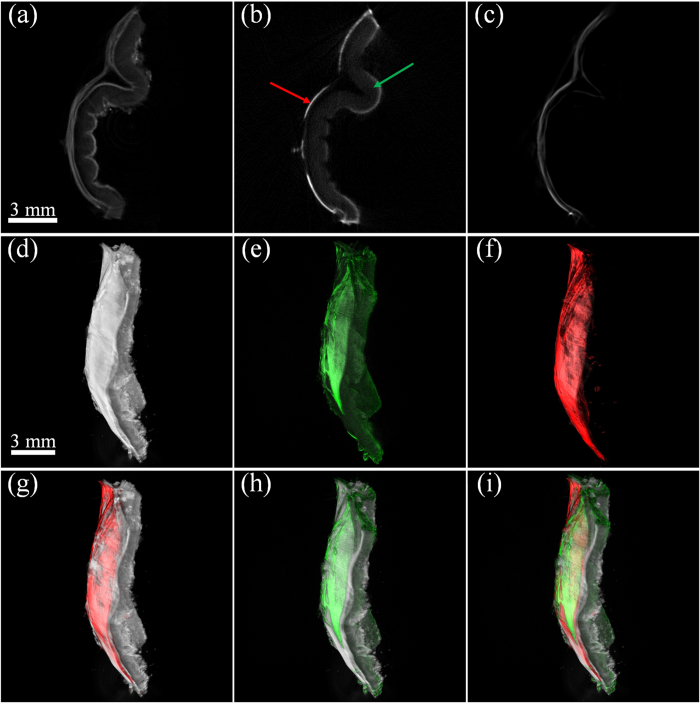
Polarization-sensitive OPT is more suitable for specific detection of the muscle fiber than tOPT and eOPT. (**a–c**) the tomographic slices of tOPT, eOPT and polarization-sensitive OPT. (**d–f**) the 3D rendering results of these three methods. (**g**) the volume rendering of the fusion image of polarization-sensitive OPT and tOPT. (**h**) the volume rendering of the fusion image of eOPT and tOPT. (**i**) the volume rendering of the fusion image of all the three methods. Green arrow: the mucosa and the submucosa. Red arrow: the muscularis externa.

Furthermore, in order to compare polarization-sensitive OPT with eOPT with muscle labeling, another sample of gastric wall was prepared by labeling the alpha smooth muscle actin with Alexa Fluor-488 dye. The sample was separately imaged by polarization-sensitive OPT, tOPT and eOPT (excitation wavelength: 488 nm, emission wavelength: 550 ± 50 nm). Tomographic images and 3D rendering results of the three methods are showed in Fig. 6(a–f), which demonstrate that tOPT provides anatomical structure of the whole sample but has no specificity for muscles. The tOPT failed to distinguished muscles because the absorption of muscle was nearly equivalent to that of the surrounding tissues after optical clearing. In contrast, eOPT highlighted the muscular tissue with fluorescence imaging. However, as shown in Fig. 6(b,e), there were artifacts in the mucosa of eOPT images, which were caused by the remaining dye or the autofluorescence. Compared with tOPT and eOPT, polarization-sensitive OPT specifically detected muscularis externa and screened out the signals of other tissues (shown in Fig. 6(c,f)). Moreover, the muscle labeling process needed two weeks more than polarization-sensitive OPT. In addition, the merged images of the three methods shown in Fig. 6(g–i) indicated that the merge of polarization-sensitive OPT and tOPT could be a promising tool for muscle imaging without tedious labeling.

## Conclusions

In this article, a polarization-sensitive Optical Projection Tomography (OPT) system with image acquisition and processing protocol was developed to enhance the detection of muscle fibers not only in a thin diaphragm section but also inside the thick gastric wall were observed with polarization-sensitive OPT. Both a commercial microscope for proof of concept and our custom OPT system illustrated that polarized images were sensitive to muscle fibers. By acquiring multi-view polarized images and adopting a Filtered Back Projection (FBP) reconstruction, tomographic imaging of muscle fibers was also achieved. Compared with tOPT and eOPT, the polarization-sensitive OPT presented a higher signal to background ratio enhancing the contrast of muscle fiber bundles. When combined with tOPT which provides general anatomic information, we have shown that greater detail of information may be provided overlaid with birefringence properties, demonstrating the potential of polarization-sensitive OPT as an 3D in-toto imaging technique for studying anisotropic tissues such myocardium, and muscle in general. In this paper, the effect of light absorption assumed constant, making use of an attenuation coefficient with a fixed value. When imaging dense and highly irregular tissues which present high attenuation values, the attenuation coefficient should be considered during the image reconstruction process. A viable way is to use the tOPT result to calculate the attenuation coefficient in each direction and correct the result of polarization-sensitive OPT.

## Additional Information

**How to cite this article**: Fang, M. *et al.* Polarization-sensitive optical projection tomography for muscle fiber imaging. *Sci. Rep.* **6**, 19241; doi: 10.1038/srep19241 (2016).
